# Clinical features of anterior blepharitis after cataract surgery

**DOI:** 10.1038/s41598-023-33956-9

**Published:** 2023-04-24

**Authors:** Tohru Sakimoto, Takeshi Sugiura

**Affiliations:** Sugiura Eye Clinic, 22 Kawanarishinmachi, Fuji-shi, Shizuoka, 416-0955 Japan

**Keywords:** Eyelid diseases, Outcomes research

## Abstract

We evaluated the clinical features of postoperative anterior blepharitis following cataract surgery and the efficacy of topical azithromycin retrospectively. Thirty eyes of 30 patients with a clinical diagnosis of anterior blepharitis by 6 months postoperatively among those who underwent cataract surgery at our institution between November 2020 and June 2022 were included. The diagnosis of anterior blepharitis and the assessment of objective and subjective findings were based on the American Academy of Ophthalmology Blepharitis Preferred Practice Pattern^®^. Azithromycin eye drops were prescribed for all patients, and findings and symptoms before and after the drops were reviewed. The time of onset ranged from 2 weeks to 6 months after cataract surgery, with the most common onset at 2 to 3 months postoperatively (mean time of onset 79.4 ± 39.6 days). The type of anterior blepharitis was staphylococcal blepharitis in 26 eyes and seborrheic blepharitis in 4 eyes, while mixed type with posterior blepharitis was noted in 6 eyes. Symptoms at the time of examination included irritation (including foreign body sensation) in 24 eyes, tearing in 4 eyes, and redness in 3 eyes. The findings and symptoms of anterior blepharitis were alleviated or resolved with azithromycin eye drops in 26 of the 30 eyes, but the blepharitis recurred in 6 of these eyes, requiring azithromycin eye drops to be re-prescribed. The onset of anterior blepharitis after cataract surgery may be related to a gradual decrease in postoperative eye drops. Patients tended to complain of irritation and foreign body sensation, and azithromycin eye drops were effective in such cases.

## Introduction

Blepharitis is a chronic inflammatory condition of the eyelids that is frequently encountered in clinical practice. The etiology of the disorder is complex and not fully understood, but the consensus is that bacteria and inflammation contribute to the pathology^1,2^. Due to its chronic conditions, permanent cure is not possible in most cases, and subjective symptoms may persist or recur even if the clinical condition has improved. Blepharitis is classified into anterior and posterior blepharitis according to anatomical classification: anterior blepharitis affecting base of the eyelashes and the eyelash follicles, and posterior blepharitis affecting meibomian glands. In the clinical classification, anterior blepharitis has traditionally been subcategorized as staphylococcal and seborrheic blepharitis, and posterior blepharitis is clinically synonymous with meibomian gland dysfunction (MGD)^1^.

Although there are no established guidelines regarding therapeutic regimens, American Academy of Ophthalmology (AAO) Preferred Practice Pattern^®^ recommends the combinations and staged treatments of a topical/systemic antibiotic, topical corticosteroid, and lid hygiene/warm compress. However, this guideline states the same treatments for all three forms (staphylococcal blepharitis, seborrheic blepharitis, and MGD) of blepharitis, which indicates that the understanding of blepharitis pathophysiology is still in its infancy.

On the other hand, it is well known that significant number of patients who undergo cataract surgery suffer postoperative discomfort and irritation, pain, dryness, burning sensation, and foreign body sensations. Various reports indicated potential involvement of dry eye diseases and MGD to those postoperative discomforts^3–5^.

Due to the improved surgical outcomes and extremely elevated patient expectations in recent cataract surgery, postoperative discomfort is often a major disappointment to the patients^6^. As stated earlier, MGD (posterior blepharitis) involvement in postoperative discomfort after cataract surgery is well documented. However, little is known regarding involvement of anterior blepharitis after cataract surgery.

In this report, we described the detail of anterior blepharitis after cataract surgery for the first time. A better understanding of the pathogenesis of anterior blepharitis after cataract surgery will allow for a more appropriate response to post-cataract surgery discomfort.

## Results

A total of 30 eyes (right eye: 18 (60%), left eye: 12 (40%)) from 30 patients were extracted. The mean age of the group was 74.9 ± 5.9 years (mean ± standard deviation), comprised of 11 males (36.7%) and 19 females (63.3%). The type of anterior blepharitis was staphylococcal blepharitis in 26 eyes and seborrheic blepharitis in 4 eyes, while mixed type with posterior blepharitis was noted in 6 eyes.

Within studied 30 patients, 28 patients had performed bilateral cataract surgery. Of these 28 patients, anterior blepharitis occurred bilaterally in 17 patients and unilaterally in 11 patients. And of these 17 unilaterally cases, 9 had the same blepharitis findings in both eyes and 11 had a difference of findings severity between the left and right eye.

**Figure 1:**
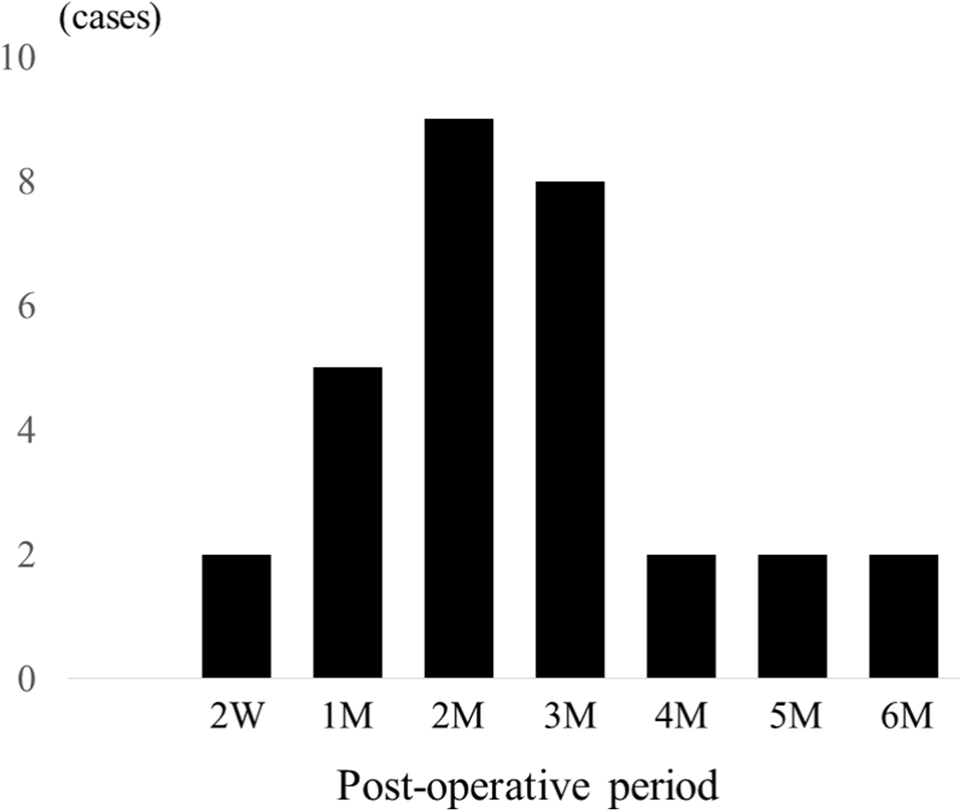
Distribution of onset period of anterior blepharitis after cataract surgery. The onset time for most of the anterior blepharitis cases was 2 to 3 months postoperatively. (W: week, M: month).

The onset period of blepharitis interviewed from patients in the medical record widely ranged from 2 weeks to 6 months after cataract surgery (mean onset period; 79.4 ± 39.6 days). Interestingly, it was concentrated between 2 and 3 months (Fig. 1).

Symptoms, which are described in AAO Preferred Practice Pattern^®^, at the time of examination were irritation (including foreign body sensation) in 24 eyes, tearing in 4 eyes, redness in 3 eyes, and photophobia in 1 eye. After topical azithromycin treatment, complete relief of symptoms were noted in 18 patients (60.0%), reduction in 8 patients (26.7%), and immutable in 4 patients (13.3%), while worsening was not noted.

In evaluation of degree of anterior blepharitis, the mean grade of eyelid debris and eyelid redness/swelling was 2.2 ± 0.7 and 1.7 ± 0.5, respectively at the time of diagnosis, and significantly reduced to 0.7 ± 0.8 and 0.4 ± 0.5, respectively after topical azithromycin treatment. Telangiectasia grade was 1.6 ± 0.7 before treatment and 0.6 ± 0.6 after treatment, which was significant reduction by topical azithromycin treatment (Table 1).

**Table 1:** Comparison of anterior blepharitis and clinical manifestations between before and after topical azithromycin treatment (mean ± standard deviation).

	Before treatment	After treatment	*p* value
Eyelid/eyelash debris grade (0–4)	2.2 ± 0.7	0.7 ± 0.8	< 0.001*
Eyelid redness/swelling grade (0–4)	1.7 ± 0.5	0.4 ± 0.5	< 0.01*
Telangiectasia grade (0–3)	1.6 ± 0.7	0.6 ± 0.6	< 0.001*

Asterisks indicates statistically significant *p*-values.

No cases required additional treatment due to failure to relieve symptoms or findings. However, recurrence of anterior blepharitis was noted in 6 cases (20%) and topical azithromycin was prescribed again. Within these 6 cases, 1 case was prescribed topical azithromycin twice (total 3 times) and another case required oral antibiotics treatment subsequently. Summary of recurrent anterior blepharitis cases in this study is described in Table 2.

**Table 2:** Summary of recurrent anterior blepharitis cases in this study.

	Number of relapses	Initial onset period of anterior blepharitis after cataract surgery	Time of recurrence	Adopted treatments
Case 1	1	1 month	1 month after initial treatment	Topical azithromycin
Case 2	1	3 months	1 month after initial treatment	Topical azithromycin
Case 3	1	4 months	1 month after initial treatment	Topical azithromycin
Case 4	1	1 month	3 months after initial treatment	Topical azithromycin
Case 5	2	2 months	5 months after initial treatment 6 months after initial treatment	Topical azithromycin topical azithromycin
Case 6	4	3 months	2 months after initial treatment	topical azithromycin
			4 months after initial treatment	topical azithromycin
			6 months after initial treatment	oral clarithromycin
			8 months after initial treatment	oral minocycline

## Discussion

It had been reported that cataract surgery can worsen blepharitis and meibomian gland function during the postoperative period to result in increased ocular discomfort and decreased vision and patient satisfaction^6–11^. However, most of these reports describe posterior blepharitis/MGD and little is known about anterior blepharitis after cataract surgery. This paper describes for the first time the clinical manifestations of anterior blepharitis after cataract surgery and the therapeutic efficacy of topical azithromycin to those patients. During the studied period, approximately 1600 cataract surgeries were performed at our hospital, and 30 of these cases showed anterior blepharitis, suggesting that the incidence of anterior blepharitis after cataract surgery is approximately 2%.

While the causes of dry eye and meibomian gland dysfunction after cataract surgery are not yet completely clear, the causes of anterior blepharitis after cataract surgery are even less understood. As far as we have been able to determine, there are no reports detailing the clinical pathogenesis of anterior blepharitis after cataract surgery. However, several causes of anterior blepharitis after cataract surgery have been postulated. First, because the patient is prohibited from washing their face for a certain period after cataract surgery (in our case, until the third postoperative day), the patients may be more susceptible to developing anterior blepharitis because of neglecting eyelid cleansing. In fact, there have been cases in which patients tend to refrain from washing their face or lid hygiene after the 3-day no-wash period because they are afraid to wash their face as they did before the surgery, resulting in the development of blepharitis. Indeed, previous report indicates the significant efficacy of lid hygiene during the perioperative period of cataract surgery^6^. However, although most of the patients had undergone cataract surgery bilaterally, the majority of them had blepharitis in only one eye or had a difference in the findings of blepharitis between the right and left eye. Therefore, the hypothesis that inadequate eyelid cleansing after cataract surgery leads to the development of blepharitis still seems uncertain. Second, our institution recommends to the patient to use topical antibiotics, topical corticosteroid, and topical nonsteroidal anti-inflammatory drug until 1 month postoperatively, but the discontinuation of these eye drops may be associated with the development of anterior blepharitis because we elucidated that anterior blepharitis develops mainly at 2 to 3 months after surgery. Third, the possibility cannot be ruled out that perioperative topical antibiotics and disinfection may have affected the commensal bacteria present in the eyelid, resulting in the development of anterior blepharitis^12^.

Regarding patients' subjective symptoms, it was interesting that most patients complained of irritation including foreign body sensation. It should be noted that most cases of anterior blepharitis after cataract surgery complain of irritation, because the fact that patients with blepharitis report irritation is often overlooked in clinical practice. Furthermore, since this was a retrospective study, we investigated in the medical records whether any of the complaints of symptoms listed in the AAO Preferred Practice Pattern^®^ had disappeared, abated, remained unchanged, or worsened. This research method is not the one used in previous reports on subjective symptoms of blepharitis in general. However, most blepharitis studies use dry eye- or MGD-related subjective symptom scores as a method of assessing subjective symptoms, which may not reflect the true symptoms of anterior blepharitis^10,11^. It is desirable to establish a clinical score specific to the evaluation of blepharitis symptoms.

The effect of topical azithromycin on anterior blepharitis that occurred after cataract surgery seems to be satisfactory. Regarding subjective symptoms, complete relief of symptoms were noted in 18 patients (60.0%) and symptoms reduction in 8 patients (26.7%). Thus nearly 90% of patients were somehow effective for blepharitis symptoms. Likewise, clinical findings were significantly diminished by topical azithromycin, indicating that topical azithromycin is leading treatment options for anterior blepharitis after cataract surgery. It should be noted, however, that although the treatment efficacy was excellent, recurrence was observed. In our study, 20% of patients required re-treatment of anterior blepharitis. Although AAO Preferred Practice Pattern^®^ clearly states that blepharitis cannot be permanently cured, certain adjunctive treatments such as lid hygiene and warm compress should be considered after cataract surgery^1^.

There is a certain limitation to this study. For example, since there are no established diagnostic criteria for blepharitis, we focused on eyelid and eyelash deposits and defined anterior blepharitis as a case with collarette or greasy deposits. Because the diagnosis was made clinically, without bacteriological examination, it is debatable whether the cases entered for suspicion of staphylococcal blepharitis were truly blepharitis or not. However, there have been many cases in which the clinical presentation closely resembles that of anterior blepharitis, even in the absence of negative bacterial cultures. Since all cases are post cataract surgery, and thus after antibiotics eye drops have been used, there should be a reduction of bacterial detection. Therefore, a focus on positive bacterial cultures in anterior blepharitis may underestimate the epidemiology of blepharitis after cataract surgery. Indeed, AAO Preferred Practice Pattern^®^ describes that diagnosis of blepharitis is based on a typical patient history and characteristic slit-lamp biomicroscopic findings, whereas microbiologic culture is positioned as an ancillary test^1^. Second, as this is a retrospective study, we are not able to specify risk factors for the development of blepharitis in any post-cataract surgery case. Additionally, because this study lacked a control group, we cannot rule out the possibility that the reduction in clinical findings observed with topical azithromycin was due to other factors such as resumption of eyelid hygiene.

This study is the first report to describe the clinical features of anterior blepharitis that occurred after cataract surgery. Anterior blepharitis mainly occurred 2 to 3 months after the surgery, tended to develop after the completion of postoperative multidisciplinary treatment. Although the recurrence of blepharitis was noted, topical azithromycin was effective to treat this clinical condition.

## Methods

Thirty patients who underwent uneventful cataract surgery in our clinic (Sugiura Eye Clinic) between November 1, 2020 and June 30, 2022, were included in the study. Patients who were diagnosed with anterior blepharitis on the operated eye at our clinic up to 6 months after the surgery, and returned within 7 to 14 days after azithromycin (Senjyu Phamaceutical, Osaka, Japan) eye drops prescription, were retrospectively examined using medical records and slit-lamp photographs. In the case of bilateral blepharitis after bilateral cataract surgery, the eye with the more severe symptoms and findings was selected as the target eye. If there was no difference between the findings of the right and left eyes, the findings of the right eye were adopted. Patients with an apparent history of blepharitis and/or eyelid surgery was excluded. Also, cases in which treatment could not be continued for any reason were excluded.

All anterior blepharitis patients were prescribed topical azithromycin (Senjyu Phamaceutical, Osaka, Japan) at the time when they were diagnosed as anterior blepharitis. Treatment regimen of topical azithromycin was 2 times daily in day 1 and day 2, and once daily from day 3 up to day 14, which were followed as instructed by the regulatory authorities in Japan.

The study was approved by the Institutional Review Board (IRB) of Sugiura Eye Clinic (No. 21000131), and all the methods described adhered to the principles of the Declaration of Helsinki. The need for written informed consent was waived by the IRB of Sugiura Eye Clinic owing to the retrospective nature of this study, in accordance with the Ethical Guidelines for Medical and Biological Research Involving Human Subjects repealed from the Japanese Government. We disclosed the study protocol to all patients and provided them with the opportunity to refuse participation.

### Perioperative treatments

Standard cataract surgery was performed with a 2.65 mm temporal corneal incision. Postoperatively, all patients were prescribed topical levofloxacin (Santen Pharmaceutical, Osaka, Japan), 0.1% fluorometholone (Senjyu Phamaceutical), diclofenac sodium (Wakamoto Phamaceutical, Tokyo, Japan) 4 times daily each from 1 day to 1 month after cataract surgery. Furthermore, nonsteroidal anti-inflammatory drug eye drops (0.1% pranoprofen, Santen Pharmaceutical, twice daily) were prescribed from the first postoperative month to 6 months. However, preoperative medications such as topical antiglaucoma and topical dry eye treatments that have been prescribed prior to surgery are basically continued. All patients were instructed to avoid getting water in the operated eyes and to not rub their eyes for 3 days after surgery.

### Subject evaluations

**Figure 2:**
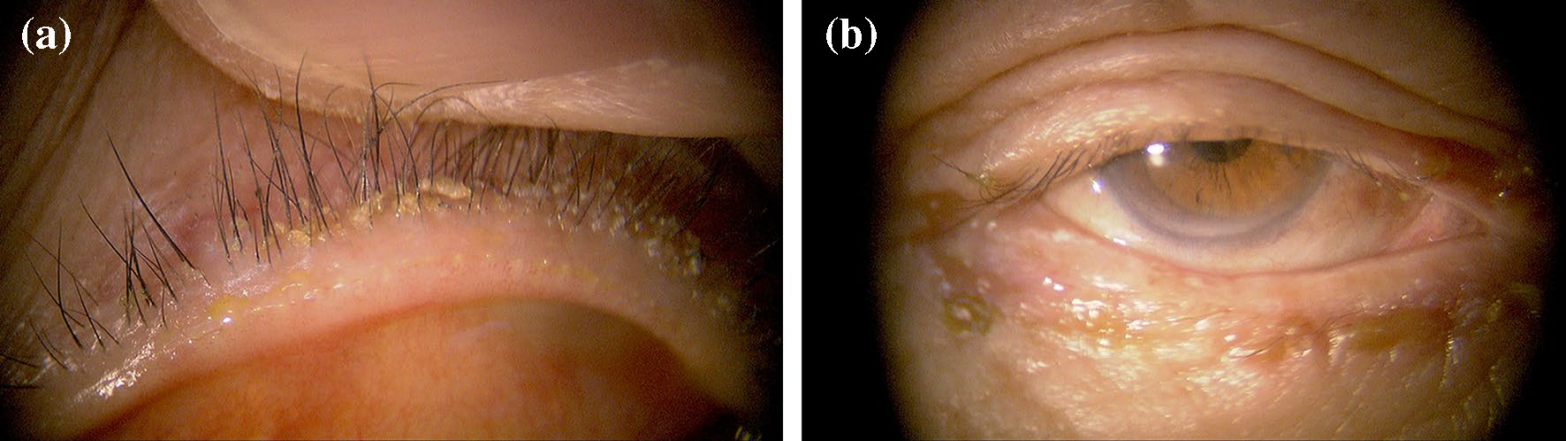
Representative slit-lamp photographs of anterior blepharitis after cataract surgery. Slit-lamp photograph of left upper eyelid margin of 70-year-old female with staphylococcal blepharitis 2 months after cataract surgery. Severe collarettes formation at the base of the cilia are noted (**a**). Slit-lamp photograph of right eye of 80-year-old male with seborrheic blepharitis 2 months after cataract surgery. Severe greasy scaling is noted in the lower eyelid (**b**).

The diagnosis of blepharitis was based on the clinical findings of eyelid deposits described in the AAO Blepharitis Preferred Practice Pattern^1^. In this guideline, it is described that staphylococcal blepharitis exhibits matted, hard scales or collarettes in their eyelid and/or eyelash, whereas seborrheic blepharitis shows oily or greasy deposits. Representative slit-lamp photographs of staphylococcal and seborrheic blepharitis are shown in Fig. 2. Furthermore, concurrence of MGD (posterior blepharitis) is also investigated. Based on slit-lamp photographs, prominent blood vessels crossing the mucocutaneous and pouting or plugging of meibomian orifices are assessed as eyelid manifestations of MGD.

Evaluations of subjective symptoms were assessed by employing symptoms and signs that are described in AAO Preferred Practice Pattern^®^ (redness, irritation, burning, tearing, itching, crusting of eyelashes, loss of eyelashes, eyelid sticking, blurring or fluctuating vision, contact lens intolerance, photophobia, increased frequency of blinking, and recurrent hordeolum) in the medical records, and after topical azithromycin treatment, patients were asked for the symptomatic relief whether they experienced complete relief, reduction, immutable, or worsening.

To evaluate the degree of anterior blepharitis, in accordance with the previous reports, slit-lamp photographs on both upper and lower eyelids were assessed. Eyelid debris and eyelid redness/swelling were graded from 0 to 4 (absent, mild, moderate, severe, and very severe). Eyelid telangiectasia was graded from 0 to 3 (no findings, mild, moderate, and severe)^6,13^.

### Statistical analysis

The clinical scores of anterior blepharitis (eyelid debris, eyelid redness/swelling and telangiectasia) before and after topical azithromycin treatment were compared using a paired t test. A *p* value of < 0.05 was considered significant.

## Data Availability

The datasets generated during and/or analyzed during the current study are available from the corresponding author on reasonable request.
